# Clinical effect of selective interventional therapy on sub-acute ST-segment elevation myocardial infarction under the guidance of fractional flow reserve and coronary arteriography

**DOI:** 10.1186/s40001-018-0319-8

**Published:** 2018-05-24

**Authors:** Bing-Jian Wang, Jin Geng, Qian-Jun Li, Ting-Ting Hu, Biao Xu, Shu-Ren Ma

**Affiliations:** 1 Department of Cardiology, Huai’an First People’s Hospital, Nanjing Medical University, No. 6 West Beijing Road, Huaiyin District, Huai’an, 223300 China; 2 Department of Cardiology, Drum Tower Hospital, Nanjing Medical University, Nanjing, 210008 China

**Keywords:** Fractional flow reserve, Angiography, ST-segment elevation myocardial infarction, Percutaneous coronary intervention

## Abstract

**Objective:**

This study aims to compare the clinical effects of selective interventional therapy (PCI) under the guidance of fractional flow reserve (FFR) and coronary arteriography.

**Methods:**

Patients with sub-acute ST-segment elevation myocardial infarction (sub-acute STEMI), who were under selective PCI treatment between April 2012 and June 2014, were included into this study. These patients were divided into two groups, based on FFR measurements: FFR-PCI group and radiography-PCI group. Then, differences in clinical symptoms, coronary angiography, intervention, and endpoint events were compared between these two groups.

**Results:**

A total of 592 patients with sub-acute STEMI were included in this study (207 patients in the FFR-PCI group and 385 patients in the radiography-PCI group). No statistical differences were observed in baseline clinical data and coronary angiography results between these two groups. Mean stent number was greater in the radiography-PCI group (1.22 ± 0.32) than in the FFR-PCI group (1.10 ± 0.29), and the difference was statistically significant (*P* = 0.019). During the follow-up period, 78 adverse events occurred (21 adverse events in the FFR-PCI group and 57 adverse events in the radiography-PCI group); and no statistical significance was observed between these two groups (log-rank *P* = 0.112).

**Conclusion:**

Selective PCI treatment in patients with sub-acute STEMI under FFR acquired similar effects, compared to PCI treatment under the guidance of radiography, which can reduce the mean stent number.

## Background

Fractional flow reserve (FFR) is an indicator to evaluate the degree of coronary ischemia, and is the ratio of lesion distal pressure and aortic pressure of the coronary artery under the maximum hyperemia by adenosine [1]. It plays a very important guidance significance in the percutaneous coronary intervention (PCI) of stable angina pectoris [2, 3] and multivessel lesions [4]. Generally, an FFR < 0.80 in coronary artery lesions is considered as myocardial ischemia in the coronary artery supply, and PCI treatment is required. However, in case of FFR ≥ 0.80, PCI treatment referred is safe and reliable [3, 4]. Currently, compared with PCI under the guidance of radiography, PCI treatment on stable and unstable angina pectoris under the guidance of FFR can partially reduce the incidence of major adverse cardiac events (MACE) [2–4], and decrease the duration of hospitalization and costs [4, 5]. In the acute phase of myocardial infarction, due to microcirculation disturbances, FFR is often higher, and the severity of the lesion may be underestimated [6, 7]. Patients with non-ST-segment elevation myocardial infarction (NSTEMI) generally receive selective PCI treatment during the recovery period. Such patients continue to benefit from PCI treatment under the guidance of FFR [4, 8, 9]. However, patients with sub-acute STEMI should receive PCI treatment in the acute phase, and FFR is not recommended for emergency PCI treatment in patients with STEMI [6, 7, 10]. A study indicated that only 30% of inpatients with STEMI received emergency PCI treatment [11], and patients with STEMI in Huai’an and other northern Jiangsu areas do not receive immediate emergency PCI treatment, but adopt selective PCI treatment due to relatively weak health awareness, short hygienic knowledge, poor economic conditions, inconvenient urban and rural traffic, and uneven distribution of medical resources. This study included patients with sub-acute STEMI who received selective PCI treatment, and compared the influence of PCI treatment under the guidance of FFR and PCI treatment under the guidance of radiography upon prognosis, in order to provide the decision basis for intervention treatment in patients with sub-acute STEMI.

## Data and methods

### Research object

Patients with sub-acute STEMI, who received selective PCI treatment in Huai’an First People’s Hospital and Nanjing Drum Tower Hospital between April 2012 and June 2014, were included into this study. Inclusion criteria were (1) diagnosis conforms to the Guideline of STEMI Diagnosis and Treatment [12]; (2) duration from the onset of myocardial infarction to PCI is ≥ 6 days [6]; (3) infract-related artery (IRA) has one lesion with ≥ 50% stenosis at least; (4) patient age is > 18 years. Exclusion criteria were (1) cardiogenic shock or unstable hemodynamics; (2) vascular conditions are poor, and PCI treatment and FFR measurement could not be carried out on vessels with a diameter of < 3 mm; (3) patients intolerant to dual antiplatelet therapy; (4) patients with adenosine contraindications; (5) IRA is the left main coronary artery or bridge vessel; (6) patients with primary cardiac myopathy; (7) patients with an expected service life of < 1 year; (8) patients with poor compliance, and do not receive regular follow-up. This study was approved by the Ethics Committee of Huai’an First People’s Hospital and Nanjing Drum Tower Hospital, and all patients provided a signed informed content. All patients were advised for FFR measurement before coronary angiography. Eventually, determining whether to measure FFR or not was decided by the patient. According to the decision whether to measure FFR or not, we divided the patients into two groups: FFR-PCI group and radiography-PCI group.

### Interventional therapy

200 μg of nitroglycerin was injected into the coronary artery, and the Judkins method was used for left and right coronary angiography. Radiography images were analyzed offline by quantitative coronary angiography (QCA) to record the stenosis degree of the lesions. For patients who agreed to receive FFR measurement, RADI Medical Systems were applied to measure FFR after coronary angiography. Pd was measured at least 3–4 cm from the distal end of the lesion by the pressure lead wire, and aortic root pressure Pa was measured by the guiding catheter. Adenosine was injected into the ulnar vein (140 μg/kg/min during at least 2 min) to acquire maximal hyperemia. At this time, FFR = Pd/Pa [3, 4]. For continuous lesions, the pressure guiding wire was slowly returned, and the lesion with the maximum pressure gradient change was selected as the target lesion [13]. For lesions in the FFR-PCI group, if FFR < 0.80, PCI treatment was carried out. If FFR ≥ 0.80, medical therapy alone was performed. For lesions in the radiography-PCI group, if stenosis degree was ≥ 70%, PCI treatment was performed. If stenosis degree was < 70, medical therapy alone was performed. Implanted stents were native drug-eluting stents, and all lesions went through pre-dilation and post-dilation before and after the implantation of the stent.

### Follow-up

After PCI, all patients received follow-up for 1 year. Endpoint events comprised MACE during the follow-up period, including cardiac death, secondary hospitalization, and secondary intervention treatment. Except for clear non-cardiac reasons, any death is considered as cardiac death. Secondary hospitalization refers to hospitalization due to angina pectoris or cardiac failure or arrhythmia, in which angina pectoris and cardiac failure can be controlled by drugs. Secondary intervention treatment refers to further intervention treatment, because angina pectoris and cardiac failure could not be controlled by drugs.

### Statistical method

Measurement data are expressed as mean ± standard deviation, and intergroup analysis adopted the independent sample *t* test. Enumeration data were expressed as the case number (percentage), and intergroup analysis adopted the *χ*^2^ test or Mann–Whitney *U* test. The survival curve is drawn using the Kaplan–Meier method for the incidence rate of MACE in both groups, and the difference between both groups was evaluated by log-rank test. Statistical software SPSS 18.0 was used for analysis. A *P* < 0.05 in both sides indicates that the differences have statistical significance.

## Results

According to inclusion and exclusion criteria, a total of 592 patients with sub-acute STEMI were included into the study. Patients had a mean age of 63.91 ± 12.15 years, and male patients accounted for 70.3%. Two hundred seven patients (35.0%) were selected to receive FFR measurement and were assigned as the FFR-PCI group, while 385 patients (65.0%) were assigned as the radiography-PCI group. No significant differences were observed in clinical situations at baseline (Table 1) and in the intergroup analysis of all indicators between both groups.

**Table 1:** Baseline characteristics of included patients

	Total (*n* = 592)	FFR-PCI (*n* = 207)	Angiography-PCI (*n* = 385)
Age (years)	63.91 ± 12.15	65.13 ± 12.74	63.26 ± 11.77
Gender			
Male	416 (70.3)	152 (73.4)	264 (68.6)
Female	176 (29.7)	55 (26.6)	121 (31.4)
Medical history			
Hypertension	298 (50.3)	113 (54.6)	185 (48.1)
Diabetes	142 (24.0)	53 (25.6)	89 (23.1)
Hyperlipidemia	49 (8.3)	17 (8.2)	32 (8.3)
Previous MI	40 (6.8)	9 (4.3)	31 (8.1)
Previous PCI/CABG	42 (7.1)	13 (6.3)	29 (7.5)
Current smokers	378 (63.9)	127 (61.4)	251 (65.2)
Body mass index	24.50 ± 3.58	24.93 ± 3.85	24.27 ± 3.41
Medicines			
Aspirin	570 (96.3)	201 (97.1)	369 (95.8)
Clopidogrel	592 (100)	207 (100)	385 (100)
Statin	592 (100)	207 (100)	385 (100)
β-Blocker	289 (48.8)	98 (47.3)	191 (49.6)
ACEI/ARB	256 (43.2)	87 (42.0)	169 (43.9)
Echocardiography			
Ejection fraction (%)	43.38 ± 6.29	43.56 ± 6.31	43.29 ± 6.27
LVEDd (cm)	5.44 ± 0.44	5.47 ± 0.41	5.42 ± 0.45

*FFR* fractional flow reserve, *PCI* percutaneous coronary intervention, *MI* myocardial infarction, *CABG* coronary artery bypass graft, *ACEI* angiotensin-converting enzyme inhibitor, *ARB* angiotensin receptor blocker, *LVEDd* left ventricular end-diastolic diameter

All patients successfully underwent coronary angiography and intervention treatment (Table 2). Mean FFR value was 0.69 ± 0.12 in the FFR-PCI group, mean duration from onset to PCI was 12.77 ± 6.63 days, and mean duration was 12.22 ± 6.34 days in the radiography-PCI group. Infract-related arteries in both groups focused on the anterior descending branch and right coronary artery. No significant statistical differences were observed in the intergroup analysis. The total number of lesions in the FFR-PCI group was 279, while the total number of lesions in the radiography-PCI group was 541. In addition, the number of implanted stents was 228 and 469, respectively. The mean number of stents in the radiography-PCI group (1.22 ± 0.32) was greater than the mean number of stents in the FFR-PCI group (1.10 ± 0.29), and the differences were statistically significant (*P* = 0.019). QCA indicated that the mean degree of stenosis for all lesions was 84.19 ± 13.24%, and no statistical differences were observed between both groups.

**Table 2:** Procedure characteristics of included patients

	Total (*n* = 592)	FFR-PCI (*n* = 207)	Angiography-PCI (*n* = 385)
Symptom to door (days)	12.35 ± 6.41	12.77 ± 6.63	12.22 ± 6.34
FFR	–	0.69 ± 0.12	–
Infarct-related artery			
Left anterior descending	274 (46.3)	98 (47.3)	176 (45.7)
Left circumflex coronary	98 (16.6)	37 (17.9)	61 (15.8)
Right coronary artery	220 (37.1)	72 (34.8)	148 (38.5)
Total lesions	820	279	541
Lesions per patient	1.39 ± 0.41	1.35 ± 0.39	1.41 ± 0.42
Stenosis (%)	84.19 ± 13.24	83.05 ± 12.04	84.78 ± 13.78
50–70%	132 (16.1)	43 (15.4)	72 (13.3)
70–90%	367 (44.8)	124 (44.4)	250 (46.2)
90–99%	321 (39.1)	112 (40.2)	219 (40.5)
Total stents	697	228	469
Stents per patient*	1.18 ± 0.32	1.10 ± 0.29	1.22 ± 0.32
TIMI grade flow	0.23 ± 0.69	0.28 ± 0.78	0.20 ± 0.64

* *P* = 0.019

**Fig. 1:**
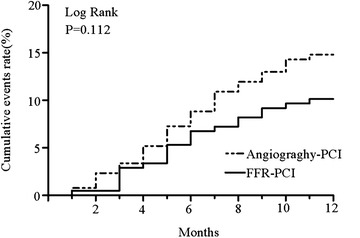
Kaplan–Meier curves of incidence of major adverse events (MACE)

After PCI, all patients received follow-up for 1 year, and 30 patients (5.1%) dropped out from the study. During the follow-up period, 78 MACEs occurred (21 in the FFR-PCI group and 57 in the radiography-PCI group), and the differences between both groups was not statistically significant. Statistical analysis was carried out for cardiac death, secondary hospitalization, and secondary PCI, respectively, and no significant difference was found (Table 3). Figure 1 shows the Kaplan–Meier curve of the incidence rate of accumulated MACEs in both groups, and the *P* value of the log-rank test was 0.112. The difference had no statistical significance.

**Table 3:** MACEs during follow-up

	Total (*n* = 592)	FFR-PCI (*n* = 207)	Angiography-PCI (*n* = 385)
Loss to follow-up	30 (5.1)	11 (5.3)	19 (4.9)
MACEs	78 (13.2)	21 (10.1)	57 (14.8)
Cardiac deaths	11 (1.9)	3 (1.4)	8 (2.1)
Rehospitalization	42 (4.7)	11 (5.3)	31 (8.1)
Repeat revascularization	25 (2.9)	7 (3.4)	18 (4.7)

*MACE* major adverse cardiac event

## Discussion

Pijls et al. [1] proposed the concept of FFR for the first time in 1993, and a large amount of clinical studies over the next 20 years have proven that PCI treatment under the guidance of FFR can acquire the same or a better effect for the treatment of critical lesions, multivessel lesions, bifurcation lesions, and left main lesions, compared to drug treatment [14–18]. DEFER [2], FAME [4], and FAMEII [3] are large randomized controlled studies that have laid the status of Class Ia indications for FFR in intervention guidelines. The DEFER study [2] included 325 patients with single vessel lesions. Patients with FFR ≥ 0.75 were divided into two groups: drug treatment group and PCI treatment group. After follow-up for 5 years, the prognosis of the former was superior to that of the latter. The FAME study indicated that PCI treatment under the guidance of FFR with a FFR cut-off point 0.80 can significantly reduce the incidence rate of endpoint events, and decrease the duration of hospitalization and costs, compared with PCI treatment under the guidance of radiography [4, 5]. FAMEII [3] divided patients with stable angina pectoris into two groups on this basis: FFR-PCI group and radiography-PCI group. After follow-up for 1 year, the incidence rate of the endpoint events of the former was significantly lower than that of the latter. The above three studies included patients with stable and unstable angina pectoris, and only the FAME study contained patients with STEMI, who received selective PCI treatment. However, no subgroup analysis was carried out.

FFR is not only affected by the stenosis degree of lesions, but is also correlated with microcirculation resistance and the number of survival myocardium in the coronary artery control area. Microcirculation resistance in the acute period of myocardial infarction is increased, the number of survival myocardium is reduced, and FFR value would be elevated; thus, underestimating the ischemic degree of lesions [19]. In addition, slow blood flow speed and heavy thrombus load of the infract-related artery in the acute phase of myocardial infarction may affect the accuracy of FFR measurement [14]. Hence, FFR measurement is not recommended in the acute phase of myocardial infarction. However, in the myocardial infarction recovery phase, when disease onset time exceeds 2 months, no significant differences are observed between the microcirculation resistance of the infarct myocardium and non-infarct myocardium [20]. Hence, in the myocardial infarction recovery phase, FFR is mainly correlated with the lesion and the number of survival myocardium. After myocardial infarction, the number of survival myocardium in the IRA control area is reduced, and the FFR value of lesions with the same stenosis degree would be higher than that in non-myocardial infarction lesions; however, ischemic degree in the IRA control area can be evaluated [10]. In addition, Pijls et al. [6] indicated that an FFR value < 0.75 in 6 days after myocardial infarction can accurately judge whether the myocardium was ischemic or not. In combination with FAME and FAMEII studies, we included patients with sub-acute STEMI who received the selective PCI treatment for over 6 days after myocardial infarction, with FFR = 0.80 as the cut-off point, in order to compare the prognosis in the FFR-PCI group and radiography-PCI group. These results indicate that no significant differences were observed in the incidence rate of MACEs between both groups during the follow-up period, while the mean number of stents in the FFR-PCI group was lower than the mean number of stents in the radiography-PCI group, indicating that FFR-PCI can lower economic burdens in patients.

Recently, the FAMOUS-NSTEMI study was published [9], in which 360 patients with NSTEMI within 5 days after disease onset were divided into two groups: FFR-PCI group and radiography-PCI group. After follow-up for 1 year, no significant differences were observed in cardiac death and the rate of secondary hospitalization between both groups. The IRA of patients with NSTEMI is the incomplete occlusion lesion. This study excluded complete occlusion lesions because the FFR of complete occlusion lesions could not be measured. QCA results indicated that the stenosis degree of lesions was mostly 70–99%, which were similar to radiography results in the FAMOUS-NSTEMI study. In addition, no differences were observed in the microcirculation resistance in the acute period of NSTEMI and stable angina pectoris [21], and no significant changes were found in the microcirculation resistance in the recovery phase of myocardial infarction, compared with that in non-myocardial infarction [20]. Hence, FFR can accurately reflect the ischemic degree of the IRA control area. This verifies the application value of FFR in selective PCI treatment for patients with sub-acute STEMI.

This study evaluates the influence of FFR-PCI and radiography-PCI on the prognosis of patients with STEMI, who received selective PCI treatment for the first time. This has guidance significance for future clinical practices and scientific researches. However, there were deficiencies in this study. First, this study is a non-randomized controlled study, and FFR-PCI and radiography-PCI are based on the selection of patients. Most of the patients select traditional radiography-PCI, and only 35.0% of patients select FFR-PCI. However, no statistical differences were observed in clinical and coronary angiography data between both groups, which increases the reliability of the conclusion to some extent. Furthermore, the mean number of stents in the FFR-PCI group was less than the mean number in the radiography-PCI group, indicating that FFR-PCI may reduce costs. Due to multiple and mixed factors, we did not directly analyze hospitalization costs.

## Conclusion

The affect of PCI treatment under the guidance of FFR was similar to PCI treatment under the guidance of radiography in patients with sub-acute STEMI. This treatment can reduce the mean number of stents, and thus, reduce hospitalization fees. However, further verification is needed through well-designed large randomized controlled studies.

## References

[1] Pijls NH, van Son JA, Kirkeeide RL, De Bruyne B, Gould KL (1993). Experimental basis of determining maximum coronary, myocardial, and collateral blood flow by pressure measurements for assessing functional stenosis severity before and after percutaneous transluminal coronary angioplasty. Circulation.

[2] Pijls NH, van Schaardenburgh P, Manoharan G, Boersma E, Bech JW, van’t Veer M, Bär F (2007). Percutaneous coronary intervention of functionally nonsignificant stenosis: 5-year follow-up of the DEFER Study. J Am Coll Cardiol.

[3] De Bruyne B, Pijls NH, Kalesan B, Barbato E, Tonino PA, Piroth Z, FAME 2 Trial Investigators (2012). Fractional flow reserve-guided PCI versus medical therapy in stable coronary disease. N Engl J Med.

[4] Tonino PA, De Bruyne B, Pijls NH, Siebert U, Ikeno F, van’t Veer M, FAME Study Investigators (2009). Fractional flow reserve versus angiography for guiding percutaneous coronary intervention. N Engl J Med.

[5] Fearon WF, Bornschein B, Tonino PA, Gothe RM, Bruyne BD, Pijls NH, Fractional Flow Reserve Versus Angiography for Multivessel Evaluation (FAME) Study Investigators (2010). Economic evaluation of fractional flow reserve-guided percutaneous coronary intervention in patients with multivessel disease. Circulation.

[6] De Bruyne B, Pijls NH, Bartunek J, Kulecki K, Bech JW, De Winter H (2001). Fractional flow reserve in patients with prior myocardial infarction. Circulation.

[7] Tamita K, Akasaka T, Takagi T, Yamamuro A, Yamabe K, Katayama M (2002). Effects of microvascular dysfunction on myocardial fractional flow reserve after percutaneous coronary intervention in patients with acute myocardial infarction. Cathet Cardiovasc Interv.

[8] Sels JW, Tonino PA, Siebert U, Fearon WF, Van’t Veer M, De Bruyne B (2011). Fractional flow reserve in unstable angina and non-ST-segment elevation myocardial infarction experience from the FAME (Fractional flow reserve versus Angiography for Multivessel Evaluation) study. JACC Cardiovasc Interv.

[9] Layland J, Oldroyd KG, Curzen N, Sood A, Balachandran K, Das R, FAMOUS–NSTEMI investigators (2014). Fractional flow reserve vs. angiography in guiding management to optimize outcomes in non-ST-segment elevation myocardial infarction: the British heart foundation FAMOUS-NSTEMI randomized trial. Eur Heart J.

[10] Pijls NH (2007). Fractional flow reserve after previous myocardial infarction. Eur Heart J.

[11] Li J, Li X, Wang Q, Hu S, Wang Y, Masoudi FA, China PEACE Collaborative Group (2015). ST-segment elevation myocardial infarction in China from 2001 to 2011 (the China PEACE-Retrospective Acute Myocardial Infarction Study): a retrospective analysis of hospital data. Lancet..

[12] Levine GN, Bates ER, Blankenship JC, Bailey SR, Bittl JA, Cercek B (2016). 2015 ACC/AHA/SCAI focused update on primary percutaneous coronary intervention for patients with ST-elevation myocardial infarction: an update of the 2011 ACCF/AHA/SCAI guideline for percutaneous coronary intervention and the 2013 ACCF/AHA guideline for the management of ST-elevation myocardial infarction. J Am Coll Cardiol.

[13] Pijls NH (2004). Optimum guidance of complex PCI by coronary pressure measurement. Heart.

[14] Kern MJ, Samady H (2010). Current concepts of integrated coronary physiology in the catheterization laboratory. J Am Coll Cardiol.

[15] Xiu J, Chen G, Zheng H, Wang Y, Chen H, Liu X (2016). Comparing treatment outcomes of fractional flow reserve-guided and angiography-guided percutaneous coronary intervention in patients with multi-vessel coronary artery diseases: a systematic review and meta-analysis. Clin Invest Med.

[16] Danad I, Szymonifka J, Twisk JW, Norgaard BL, Zarins CK, Knaapen P (2016). Diagnostic performance of cardiac imaging methods to diagnose ischaemia-causing coronary artery disease when directly compared with fractional flow reserve as a reference standard: a meta-analysis. Eur Heart J.

[17] Nascimento BR, Belfort AF, Macedo FA, Sant’Anna FM, Pereira GT, Costa MA (2015). Meta-analysis of deferral versus performance of coronary intervention based on coronary pressure-derived fractional flow reserve. Am J Cardiol.

[18] Zhang BC, Zhou ZW, Wang C, Ma YF, Li WH, Li DY (2014). Fractional flow reserve improves long-term clinical outcomes in patients receiving drug-eluting stent implantation: insights from a meta-analysis of 14,327 patients. Int J Cardiol.

[19] Kern MJ (2011). To catch a thief: opening a CTO and its effect on the contralateral donor artery FFR. Cathet Cardiovasc Interv.

[20] Marques KM, Knaapen P, Boellaard R, Lammertsma AA, Westerhof N, Visser FC (2007). Microvascular function in viable myocardium after chronic infarction does not influence fractional flow reserve measurements. J Nucl Med.

[21] Layland J, Carrick D, McEntegart M, Ahmed N, Payne A, McClure J (2013). Vasodilatory capacity of the coronary microcirculation is preserved in selected patients with non-ST-segment-elevation myocardial infarction. Circ Cardiovasc Interv.

